# Research on the Evaluation of Reformation and Revolution System for Universities Based on Neural Network

**DOI:** 10.1155/2022/6143259

**Published:** 2022-06-09

**Authors:** Ning Ma, Zhifeng He

**Affiliations:** ^1^Ordos Vocational College, Ordos017000, China; ^2^School of Government and Law, Beijing Normal University, Zhuhai519087, China

## Abstract

Accurately evaluating the working conditions of college revolution and reformation system personnel is currently a hot issue in the field of revolution and reformation system research. Based on the neural network architecture, this paper constructs a model for the system and obtains relevant quality evaluation indicators through simulation experiment analysis. According to the quality evaluation index framework of the revolution and reformation system, the experimental platform selects 7 universities in the region, 6 of which are samples, and 1 (S University) is the research target, and the scores of each index are calculated using MATLAB auxiliary software. The simulation process starts from the characteristics of the actual neural network model; selects 17 evaluation impact indicators, including 3 basic indicators and 14 technical indicators based on historical data; and uses the factor recursive method to improve the neural network and establish an evaluation model. Then use the collected specific data to train a BP neural network with a structure of 21 × 11 × 1, continuously adjust the weights and thresholds of the neural network until the standard error requirements are met, finally verify the scientificity of the evaluation model, and compare the actual output value with the expected output value for comparison. The experimental results show that the model input data redundancy rate is reduced to 0.136, and the network training time delay is reduced to 413 ms, which improves the computing power of the network model of the reformation and revolution system in colleges and universities. The use of neural network data to reduce dimensionality effectively promotes the reformation and revolution system in universities.

## 1. Introduction

The reformation and revolution ability of students of related majors for universities has gradually become an indicator for the quality of training related majors. More and more scholars have begun to focus on cultivating the innovative spirit and entrepreneurial ability of students in related majors for universities [1]. However, reformation and revolution education started very late in the region, and a systematic reformation and revolution evaluation system has not yet been formed. The research on reformation and revolution evaluation of related majors is particularly scarce. At present, the education field in the area urgently needs to solve the problem of the development of related majors in colleges and universities. The quality of educational reform is a matter of scientific evaluation. The purpose of this topic selection is to enrich the theoretical methods of reformation and revolution education evaluation of relevant majors. Taking S University as a research sample, a relevant evaluation index system is set, and a neural network-based reformation and revolution education evaluation model is established on the basis of it. The evaluation and training of relevant professional reformation and revolution education provide a relevant basis. By expanding the theoretical methods of relevant professional reform and reform education evaluation, the relevant professional reform and reform evaluation indicators are analyzed and screened. At the same time, combining the status quo and investigation to determine the reliability of the model provides a new perspective for the evaluation field [2–4].

With the development of deep learning technology, more researches have begun to pay attention to the application of deep learning technology in recommender systems. In terms of practical applications, although the number of projects on the entrepreneurial project information platform continues to increase, causing a serious information overload to users, the application of the entrepreneurial project recommendation system has not kept up and only stays at the stage of non-personalized recommendation. Quantitative analysis is the guarantee of scientific research. It mainly uses relevant software for scientific calculation. According to the calculation results, it finds out the deficiencies in the process of reformation and revolution education in relevant colleges and universities and puts forward corresponding suggestions for improvement [5–8]. Entrepreneurial reformation quality evaluation has the potential to motivate the evaluation object to achieve the entrepreneurial and innovative goals, that is, it can guide colleges and universities to strive to achieve the predetermined entrepreneurial and innovative goals, promote the optimization of the entrepreneurial reformation process, improve the quality of entrepreneurial reformation and school-running efficiency, and is conducive to the administration of entrepreneurial reformation. This guiding effect is generally achieved by increasing or decreasing the weight of the index in the index system [9–11].

This paper researches the quality evaluation in the social science entrepreneurial reformation system for universities by combining theoretical research with empirical research. Firstly, the research background and purpose of the paper are introduced; secondly, according to the setting principles of comprehensive conciseness, objective comparability, and operability, the evaluation index system of the social science revolution and reformation system for universities is constructed. This paper uses SPSS software to carry out a validity analysis of the data collected by the questionnaire. The analysis shows that the factor loading value and KMO value are all greater than 0.5, the significance test results are all less than 0.01, and the explanation degree of each variable is more than 60%. It indicates that each option has a significant degree of distinction, and the structure meets the relevant requirements. Compared with the traditional non-machine learning or simple technical index analysis, the multivariate non-linear system model has a good ability to deal with this problem and has a more convenient and effective application significance for the prediction research of entrepreneurial reformation quality evaluation coefficient. However, because the neural network itself still has some defects, such as the problem of data redundancy in the input data, it is difficult to find the global optimal value, which is often the local optimal value, and there are also problems such as too long training time. Therefore, based on the shortcomings of the neural network mentioned above, this paper proposes the PNN model. This combined model can be regarded as a derivative model of the neural network, which not only greatly optimizes the network performance but also significantly improves the quality evaluation of entrepreneurial reformation to predict the effect.

## 2. Related Work

There will be new requirements for information acquisition, processing and transmission problems, network routing optimization problems, data security, confidentiality issues, and so on, which will become the primary task of social operation. Therefore, neural computing and evolutionary computing and high-speed information network will be closer and will play a huge role in the field of computer networks. Neural network theory has achieved remarkable results in computing theory, forming new concepts of neural computing and evolutionary computing, which has aroused the strong interest of many theorists. However, with the continuous expansion of the scale of the entrepreneurial project information platform, it is more difficult for users to browse all entrepreneurial projects completely and quickly find the projects they are interested in, causing a serious information overload problem for users. In reality, the recommendation system is usually used to alleviate the problem of information overload. It allows users to obtain the information they need from a large amount of information in a short period of time, reducing the impact of information overload, thereby improving the user experience of the information platform [12–14].

For the concept of relative efficiency, Sun et al. [15] made an efficiency evaluation on the evaluation object and comprehensively evaluated the efficiency situation. Because of its practicality and the advantage of not requiring any weight assumptions, this research method has been well promoted and widely used in a very short period of time. It is only suitable for comprehensive evaluation among similar units, and the model requires that the number of units should be much larger than the number of indicators. Otherwise, it will lead to relatively effective consistent attributes of most or even all decision-making units. situation, no precise conclusions can be drawn. Neural networks have more inputs to learn and store. Mahdi et al. [16] argue that the ability to output schema mappings does not require prior disclosure of the mathematical equations describing such mappings. This method follows the rules of the gradient descent method and uses backpropagation to gradually adjust the weights. In the process of revolution and reformation quality assessment, network and computer technology were not fully utilized. Talukder et al. [17] designed a management information system for revolution and reformation quality assessment for universities to automate the work of revolution and reformation quality assessment, including manual form filling, manual auditing, and manual scoring. The evaluation work efficiency is low; the accuracy is poor; and it is not conducive to the in-depth information analysis work.

It is also the main function of the neural network that urgently needs to be enhanced. Han et al. [18] further studied the algorithm for adjusting the multilayer perceptron, making the established model and learning algorithm a powerful tool for adaptive neural network, and constructing a composite network of the multilayer perceptron and self-organizing feature map-level association. Chen et al. [19] first introduced the recommendation process, recommendation principle, and recommendation result generation method of the above two algorithms in detail. Then the program is implemented according to the principle of the algorithm, and offline experiments are carried out using the entrepreneurial project data set collected from the Internet. The experimental results are compared with PMF, ConvMF, and other algorithms. The experimental can better complete the task of entrepreneurial project recommendation, and effectively solve the problems between users and projects. In terms of academic research, Li et al. [20] found that recommender systems are still a research hotspot; more studies have begun to focus on the application systems. In terms of practical applications, although the number of projects on the entrepreneurial project information platform continues to increase, causing serious information overload to users, the application of the entrepreneurial project recommendation system has not kept up and only stays in the stage of non-personalized recommendation. Therefore, the research problem of this paper is not only the theoretical value but also has certain practical significance [21–25].

## 3. Layering of Iterative System Based on Neural Network

### 3.1. Neural Network Vector Analysis

It is a multilayer feedforward type network in the neural network, which is very powerful in non-linear mapping ability. The difference between various neural network models is that their transformation functions are different, thus forming different information processing characteristics. The transformation function of the neuron reflects the relationship between the activation state, the inhibition state, and its output of the neuron.

(1) $$\begin{array}{c} \left\{\begin{array}{c} \sum \frac{1}{2}W{\left(i,j\right)}^{t}W\left(i,j\right)+\text{theta}\left(i,j\right)=0,\\ \sum \frac{1}{2}s{\left(i,j\right)}^{t}s\left(i,j\right)=1. \end{array}\right. \end{array}$$

Through the training and learning of known samples, the network output is constantly approached to the expected output under the condition of constantly changing the weights and their topology. We call this process the training or learning of the neural network. Variable weights are dynamically adjusted. The rules for changing the weights are called training algorithms or training rules and are sometimes called learning algorithms or learning rules. The operation of the neural network generally includes two periods of learning and work: the purpose of the learning phase is to extract the rules and knowledge implicit in the training data through learning and store it in the network for use in the subsequent work phase.

(2) $$\begin{array}{c} \text{delta}\left(t,j\right)=\left\{\begin{array}{c} \text{fig}\left(\text{net}\left(t,j\right)-\text{sigma}\left(t,j\right)\right),\\ \text{fig}\left(\text{net}\left(t,j\right)-\sum \frac{1}{2}s{\left(t,j\right)}^{t}s\left(t,j\right)\right). \end{array}\right. \end{array}$$

Outside of this learning style, there is a “college” that can provide the expected output conclusion (i.e., the correct answer). Then this will continuously compare its actual output with the expected output. If the two do not match, it will adjust the weights according to certain rules according to the range of errors to ensure that the actual output value will gradually increase, close to expectations, which is conducive to jumping out of the local minimum, but the training speed of the algorithm will still be slow. It shows that the model has generated a better prediction function relationship after the training set fitting optimization. Only when a high-quality prediction function is obtained in the training phase can the prediction output result be accurate and effective.

(3) $$\begin{array}{c} \text{fig}\left(\text{net}\left(s,t\right)-\text{sigma}\left(s,t\right)\right)=\frac{1}{\exp\left(-\text{net}*f\left(s,t\right)\right)-1}-\frac{1}{\exp\left(-\text{net}*f\left(s,t\right)\right)+1}. \end{array}$$

According to the content of the innovation and entrepreneurship education evaluation system for computer majors, an education evaluation model based on a neural network is constructed. First, the 21 evaluation index scores that affect the innovation and entrepreneurship education of computer majors are used as the input vector of the neural network, and the evaluation result is used as the output vector. The fitting error and prediction error of the dynamic neural network are generally larger, especially the deviation between individual prediction points and test points is relatively large, indicating that the accuracy of the prediction results of individual points is not good, but if you consider the ups and downs of the entrepreneurial reformation system market, the volatility characteristics of the dynamic neural network are still quite good, and it has a good prediction effect and reference value. On the basis of the traditional gradient descent method, a value is added to the adjustment of each weight (or threshold) this time and descends according to the gradient method to generate a new weight (or threshold) change.

### 3.2. Network System Hierarchical Settings

**Figure 1:**
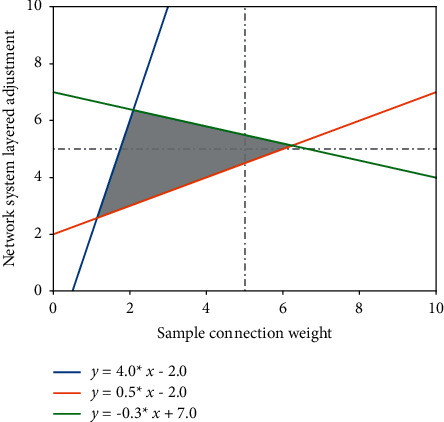
Adjustment of hierarchical connection weights in the network system.

The neural network learning algorithm essentially takes the sum of the squares of the network errors as the objective function and uses the gradient method to find the objective function to reach the minimum value. Adjust and modify the connection weights of the network to minimize the error modulation. There are two processes of learning process calculation and error backpropagation. Therefore, a better way is to combine these two algorithms to form a new algorithm. It can take into account not only the advantages that the adjustment amount of the connection weight of the neural network is determined but also the randomness and tentativeness of the adjustment (amount) of the connection weight in the simulated annealing algorithm. Therefore, the combination of the neural network and simulated annealing algorithm can be realized by dividing the modification of a connection weight into two parts: the direct calculation part is provided by the neural network in Figure 1, and the random part is provided by the simulated annealing algorithm.

The sample set should be as representative as possible. In order to accurately fit various sample data, the system finally obtains potential patterns through hundreds or even thousands of training and learning. When it encounters new sample data, the system automatically makes predictions and classifications based on the training results. In the gradient descent method with adaptive *r*, the basic idea is that the learning rate is constantly self-correcting according to the change of the error so that the weight coefficient is continuously adjusted in the direction of reducing the error. Increasing the learning rate within a certain range usually converges faster than standard gradient descent algorithms. However, when *v*(*w*(*t*)) is small, the learning rate decreases because the weight correction of the algorithm is still small.

(4) $$\begin{array}{c} \sum \frac{\text{net}\left(i,j\right)-\text{sigma}\left(i,j\right)}{\text{net}\left(i,j\right)+\text{sigma}\left(i,j\right)}=\sum_{i=1,j=1}^{i,j}\frac{w\left(i,j\right)}{w\left(i,j\right)-1}. \end{array}$$

When the input function value is large, its slope will be close to zero, resulting in a very small gradient change, which makes the adjustment process of the network weights almost stagnant. Elastic methods often ignore the magnitude of the partial derivative and only take the sign of the partial derivative. The sign in the formula is a sign function. When the network training oscillates, the adjustment amount of the weights decreases; when the weights all change in the same direction in several iterations, the adjustment amount increases. The algorithm converges faster than the previous methods without consuming more memory. First, summarize the cases and then select the cases and content coding. The first step is to split and merge the phrases in the cases. The second step is to eliminate objections between phrases and unify opinions. The third step is to analyze and verify again in order to ensure the comprehensiveness, accuracy, and objectivity of the indicators. See the text for the content of the final index element extraction.

### 3.3. Iterative System Feature Calculation

Reformation and revolution education requires multiorganization and coordinated operation, and comprehensive analysis of professional characteristics, student characteristics, campus infrastructure, and other factors and select specific samples determine to collect the corresponding evaluation results, divide them into training samples and test samples, and conduct simulation learning through MATLAB to check the calculation results of the model; if the error is within the allowable range, the model can be used to evaluate the output value with the corresponding level delineated before the evaluation. In the principal component analysis (PCA) dimensionality reduction stage, after repeated attempts, it is found that the 99% principal component ratio is chosen to reduce the dimension, which is the best ratio. After the principal component dimension reduction, the dimension of the data is reduced from 37 dimensions to 11 dimensions.

(5) $$\begin{array}{c} \text{findtest}\left(u,v\right)=\langle \begin{array}{c} u-v,\;\text{if}\left[u-v<1\right],\\ u+v,\;\text{if}\left[u-v>1\right]. \end{array} \end{array}$$

Then, enter the stage of the coefficient group optimization algorithm (PSO) debugging to find the optimal parameters. After repeated attempts, comparisons, and adjustments, the set parameters are obtained. The optimized coefficient group optimization parameters are specifically set as, and the population evolution algebra is 200 generations with a population of 40. It has been tested and verified that the RBF kernel function selected by the support vector machine has the best prediction result. Therefore, after data processing, input the support vector machine model with RBF as the kernel for sample training to obtain the prediction function and predict the output result.

(6) $$\begin{array}{c} w\left(a\left(i,j\right),b\left(i,j\right)\right)=\left[\begin{array}{c} \text{findtest}\left(\text{netu}\left(i,j\right),\text{netv}\left(i,j\right)\right)\\ \text{sigma}\left(i,j\right) \end{array}\right],\;\left\{i,j=0,1,2,\ldots ,n\right\}. \end{array}$$

The innovation and entrepreneurship education evaluation system for computer majors covers 4 first-level indicators and 21 second-level indicators. The innovation and entrepreneurship education evaluation system also determines the network structure of the education evaluation model. The characteristics of distributed storage of information and parallel computing make the network show good associative memory function and fault tolerance, and the damage of some neurons will not affect the overall performance of the network. The prediction error in Table 1 of the network is *E* = *T* − *Y*.

It is generally believed that a large number of neurons in the hidden layer can take advantage of their decentralized storage to process various complex information and greatly improve the running rate of the entire neural network by improving the work efficiency of a single neuron. But, on the other hand, the disadvantage of increasing the number of nodes is also obvious, that is, it will make the model structure too complicated, increase the burden of model training, and even cause overfitting. Therefore, in practical applications, the choice of the number of hidden layer nodes has a huge impact on the prediction effect of the final model. Before performing neural network analysis on the data, it is necessary to normalize the data into a number between the interval [0, 1]. The significance of normalizing the data is to eliminate the dimensions of different index data, avoid the results of model prediction failures, and at the same time improve the running speed of the model.

**Figure 2:**
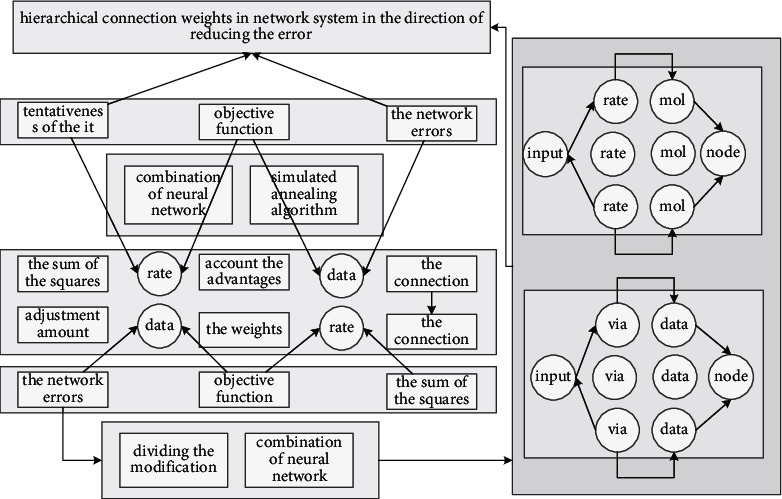
Network structure of the entrepreneurial reformation quality assessment model.

The article refers to the evaluation index system of revolution and reformation work level of ordinary colleges and universities and strives to reflect the principles of scientificity, comprehensiveness, accuracy, and measurable operability. The index system in Figure 2 is designed as the following 6 first-level indicators. These 18 secondary indicators fully reflect the reform situation. First of all, as a computer application system, it should incorporate some basic contents in the evaluation of entrepreneurial reformation quality to collect evaluation information, process evaluation data, and evaluate results and provide a good user interface and other functions.

### 3.4. Principal Component Analysis of Network Nodes

**Figure 3:**
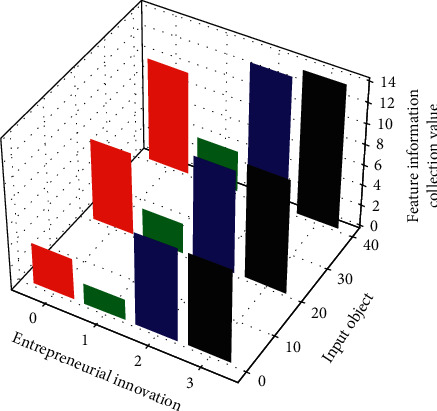
Collection of characteristic information of entrepreneurial reformation objects.

Since the input of the secondary indicators of the network nodes is obtained by students scoring by using the percentage system, the magnitudes of the values of each component are quite different. The overall structure of the model is basically determined, and no significant adjustment is made to it, so some of the structural parameters are constant, which are given in the first line of it. The parameters that need to be set are the number of multiscales *M*, the void ratios *d*_1_, *d*_2_,…, *d*_*M*_ at each scale, and all possible combinations of multiscale ratios *W*. When implementing the method, first select a possible multiscale scale *w* in *W* and calculate the undetermined hyperparameters in the MFCN module at this scale. By setting a number *N*, a specific model structure can be determined. Under this structure, the training set *D* can be used to train MFCN, and the test set *T* can be predicted; the best results in the above structure exploration process are retained as the model's *M* scale as shown in Figure 3.

This paper mainly focuses on students' background, students' professional ability, students' practical ability, and students' development ability. Because the Delphi method has a significant role and significance in the construction of evaluation indicators, the Delphi method is used to create an evaluation of innovation and entrepreneurship education for computer students. In the index system, the contents of the evaluation index of innovation and entrepreneurship education are shown as follows. The lower part represents the deep neural network, where the input is the feature of the entrepreneurial project, and the output is the latent feature of the project, which is used to extract the latent feature of the project.

(7) $$\begin{array}{c} \text{flabel}\left(W^{t}W+b\right)=\sum_{i=1,j=1}^{i,j}\sqrt{{\left(\frac{w{\left(i,j\right)}_{d-1}^{d+1}}{W^{t}W}-\frac{w\left(i,j\right)}{W^{t}W-1}\right)}^{2}+1}. \end{array}$$

The existence of the ARCH effect in the sequence is a necessary premise for its modeling using the GARCH model, so the ARCH effect test of the sequence data is also a crucial step. It can be seen that each neuron in the input layer receives a model indicator, and finally, the output layer outputs the daily closing price of the entrepreneurial reformation system index. The five neurons in the middle layer are connected to the input and output layer neurons. There are different values connected between them, which are the connection weights between different layers in the neural network. This paper implements this step using the LM test, and the function used is the ArchTest() function in the R software. The test results show that the *P* value is 5.885*e* − 09, which is close to zero. At this time, the null hypothesis is rejected, thus verifying the existence of the ARCH effect in the return series, and then we can use the GARCH model to fit the return series.

## 4. Construction of Evaluation Model of Reformation and Revolution System for Universities Based on Neural Network

### 4.1. Neural Network Data Preprocessing

Combined with the quality evaluation index system of the social science revolution and reformation in colleges and universities, 21 third-level index data in the system are selected as vectors. To find the minimum deviation, the most common method is the optimal gradient descent method. The verification experiment of DS-NN is first implemented on the UCR standard time series classification data set, and the performance is compared with other baseline models to prove the effectiveness of DS-NN on the TSC task; in addition, because this model is suitable for other one-dimensional sequence data, try to use DS-NN to solve the actual traditional sequence data classification problem aero-engine fault diagnosis. Both experiments were performed according to the procedure described in the four stages. After demonstrating and analyzing the effectiveness of the proposed model on standard data sets and practical application problems in similar fields, it will further analyze the interpretability of the model and the construction of evidence bodies based on the data of the two experiments above. The proposed improvement method is effective.

(8) $$\begin{array}{c} \langle \begin{array}{c} \sqrt{\frac{{\left(w{\left(i,j\right)}_{d-1}^{d+1}-w\left(i,j\right)\right)}^{2}}{w{\left(i,j\right)}_{d-1}^{d+1}}}\\ \sqrt{\frac{{\left(w{\left(i,j\right)}_{d-1}^{d+1}+w\left(i,j\right)\right)}^{2}+1}{w\left(i,j\right)}} \end{array}\rangle =1. \end{array}$$

Classroom teaching quality evaluation is the core part of the teaching quality evaluation system in colleges and universities. It consists of three modules, namely sample database maintenance, neural network training, and neural network evaluation. In order to ensure the safe use of the software, the login interface of the system is designed. If the user name and password cannot be entered accurately for three consecutive times, the system will be logged out. We can see that the ACF and PACF fluctuations of the residual series and residual squared series do not exceed the corresponding confidence intervals, indicating that there is no autocorrelation in the two time series in Table 2. From this point of view, it is an appropriate choice to apply the GARCH model to the index return sequence of the entrepreneurial reformation system.

In the entrepreneurship major, the outstanding performance of students' professional ability is to obtain the advanced qualification certificate in the software level examination. At the same time, according to the data collected by the author, the proportion of students who obtained the advanced qualification certificate in the computer technology and software professional and technical qualification examination is quite small, indicating that the advanced qualification certificate of the software proficiency examination has a heavyweight. From the data analysis collected by the school, the professional knowledge of the school's major students is relatively solid.

(9) $$\begin{array}{c} \Delta w\left(i,j\right)-a\frac{\text{d}f\left(i,j\right)}{\text{d}w\left(i,j\right)}-b\sqrt{\frac{\text{d}f\left(i,j\right)}{\text{d}w\left(i,j\right)}}-c=0. \end{array}$$

For the evaluation of entrepreneurial reformation quality, it can be regarded as a non-linear mapping from input (evaluation index of entrepreneurial reformation quality) to output (final evaluation result of entrepreneurial reformation quality in colleges and universities). If we choose too few hidden layer nodes, the convergence speed of the entire neural network will be slow and difficult to converge. On the contrary, if we choose too many hidden layer nodes, it will cause the topology of the neural network. Complex iterative learning requires a large amount of computation, and the error is not necessarily the best. In addition, too many hidden nodes will increase the training time.

### 4.2. Evaluation and Prediction Scale of Reformation and Revolution System

**Figure 4:**
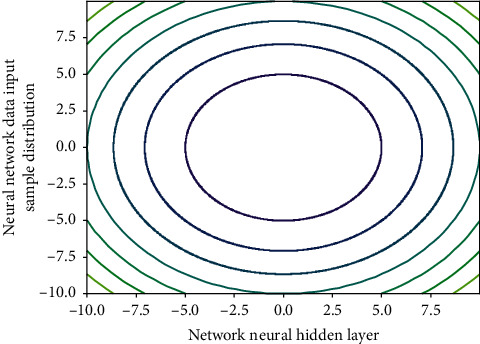
The distribution of neural network data input samples.

The first is to organize the data source: this module adopts B/S mode; it can be observed that in terms of average precision and the number of times achieving the best performance (including winning alone), in the single model, the deep learning method performs well and is better than the classical non-deep learning method. On the above data set, the overall accuracy of the method is about 94%, which is higher than the BOSS method. The reason is that the global distance between samples or features that some non-deep learning methods rely on cannot reflect the real difference between samples. Since there are many students grading in each class, it is equivalent to having many judges. Therefore, in this paper, the five highest scores and the five lowest scores are removed to take the average value as the value of the 16 input indicators of the teacher; thus, the factors that some students are irresponsible in evaluating teachers can be eliminated. The output index is the evaluation value of classroom teaching quality. This paper adopts the evaluation value of the teaching supervision group after listening to the classroom and uses such sample data to train the network. Inverse normalization processing is performed to obtain the predicted value of the neural network, and finally, error analysis in Figure 4 and result prediction are carried out.

The database system of B/S (browser/server mode) uses JSP as the middle layer of database operation and closely combines the database structure of client-server mode with Web technology, thus forming a three-tier Web structure. The B/S mode decomposes the server part in the traditional C/S mode into a database server and one or more application servers (Web servers), thus forming a client-server system with a three-tier structure. The B/S architecture can be divided into it, as presentation layer (client layer), functional layer (application server layer), and data layer (database server layer).

(10) $$\begin{array}{c} \left\{\begin{array}{c} \frac{\text{d}f\left(i,j\right)}{\text{d}w\left(i,j\right)}⟶\sqrt{\frac{\text{d}f\left(i,j\right)}{\text{d}w\left(i,j\right)}},\\ \sum_{i=1,j=1}^{i,j}\frac{1}{i-j}\sqrt{{\left(w{\left(i,j\right)}_{d-1}^{d+1}-w\left(i,j\right)\right)}^{2}+1}⟶w{\left(i,j\right)}_{d-1}^{d+1}. \end{array}\right. \end{array}$$

In order to select the best fitting model, this paper adopts the four models such as GARCH (1, 1), GARCH (1, 2), GARCH (2, 1), and GARCH (2, 2) for the index return of the entrepreneurial reformation system. The time series data are fitted simultaneously. For the selection of the best fitting model, we usually comprehensively consider the three evaluation indicators of AIC value, BIC value, and maximum likelihood value. Among them, the AIC value is similar to the BIC value, and the smaller the value is, the more effective the model is. On the contrary, the maximum likelihood value is the opposite. The larger the value, the smaller the error of the model prediction result, and the better the model. The goodness of fit of the model does not get better as the number of neurons in the hidden layer increases. When the number of nodes in the hidden layer of the neural network is 5, 8, and 10, the model fitting effect is better than other models. When the number of nodes is 5, the root mean square error (RMSE) and the absolute value of the error percentage MAPE reach the lowest, which are 23.4417 and 0.0087, respectively, and the prediction effect of the neural network is the best at this time.

(11) $$\begin{array}{c} a\frac{\nabla f\left(i,j\right)}{\nabla w\left(i,j\right)}*\sqrt{a}\frac{\nabla^{1/2}f\left(i,j\right)}{\nabla w^{1/2}\left(i,j\right)}*\cdots *\sqrt[{n}]{a}\frac{\nabla^{1/n}f\left(i,j\right)}{\nabla w^{1/n}\left(i,j\right)}=1. \end{array}$$

The size of the eigenvalue of each original indicator is proportional to the amount of information it covers. Usually, we take “eigenroot greater than 1” as the basis for selecting principal components. As the most effective empirical method among the current principal component analysis methods, the characteristic root method is also the method most frequently used by researchers. Considering that the characteristic root method is only a rule of thumb, there will be occasional errors, which may overestimate or underestimate the actual number of principal components, so it is usually necessary to combine other methods such as gravel diagrams for comprehensive evaluation. This paper hopes that the input variables will cover the information of the original data as comprehensively as possible, so the cumulative contribution rate is selected as 90%. For the number of principal components above, a total of four principal components were selected.

### 4.3. Evaluation Model Dynamic Scale Analysis

After selecting a suitable sample, the sample data must be normalized before being input to the ANN network because, after the above data collection, it can be seen that the values of various indicators of sample input and output are large or small. The values are far from each other, so the indicators must be normalized to prevent the information of small values from being overwhelmed by the information of large values. It can be seen that the selection of the initial weight has an important influence on the training of the neural network. We know that in the early stage of neural network training, it is often necessary to set the connection weights of the network to some small random numbers, which can make the weighted sum of the input values of each neuron in the network smaller, and work in the place where the slope of the activation function changes the most, and at the same time, it also prevents the unreasonable and infinite growth of the absolute value of some weights after continuous learning many times. For this reason, in the neural network model for the quality assessment of entrepreneurial reformation, we take different small random numbers for the initial weights for many times during the parameter setting process in the early stage of network training and train separately. Then this paper compares the training performance in Table 3 and finally outputs the random number set with the smallest error.

When developing the entrepreneurial reformation quality assessment system, this paper chooses SQL Server 2010 as the database tool and uses the neural network application module developed by Microsoft Visual Basic 6.0 to process the relevant data of the entrepreneurial reformation quality assessment, and the processing results are also stored in SQL Server 2010, which is a true relational database management and analysis system designed for creating scalable e-commerce, online commerce, and data warehousing solutions. Not only does SQL Server 2010 work effectively as a powerful database server, but the database engine is also used in applications that require independent databases to be stored locally on the client. SQL Server 2010 can dynamically configure itself to efficiently use the available resources of client computers.

(12) $$\begin{array}{c} \frac{a}{\nabla f\left(i,j\right)-a}-\frac{\nabla f\left(i,j\right)}{\nabla w\left(i,j\right)}=\left\{\begin{array}{c} \text{sigma}\left(i,j\right)-1,\\ \left(\text{sigma}\left(i,j\right)-a\right)f^{\prime }\left(\text{net}\right). \end{array}\right. \end{array}$$

The frequency of index elements is of great significance to the selection. The frequency of evaluation indicators such as “entrepreneurship education,” “subjectivity education,” and “personalized education” in the 35 literatures are all below 20, which is significantly lower than the frequency of the other 24 evaluation indicators. The methods and perspectives of the indicator system are different and meet the differences and coverage. Therefore, these 24 indicators can have certain accuracy and comprehensiveness, and the selection of indicators is more reasonable. Consideration should be given to reforming the three-level indicators of the quality evaluation indicator system. In this study, through expert opinion consultation, a total of 32 index consultation forms for the system were distributed, and a total of 11 valid questionnaires were recovered, with a recovery rate of 35.48%.

(13) $$\begin{array}{c} \overset{i,j}{\overset{︷}{\frac{1}{2}s{\left(i-n,j\right)}^{t}s\left(i-n,j\right)+\frac{1}{2}s{\left(i-n+1,j\right)}^{t}s\left(i-n+1,j\right)+\cdots +\frac{1}{2}s{\left(i,j\right)}^{t}s\left(i,j\right)}}⟶\lim\left(i,j\right). \end{array}$$

The basic probability distribution when two evidence bodies make the same decision is shown in the text. By comparing the basic probability distributions of the given evidence bodies, it can be found that when two evidence bodies give the same judgment, the probability value of the uncertainty item in the basic probability distribution is very small, but when the evidences conflict, the value of this item is very large. According to the formula, the probability of uncertain items will simultaneously be a part of the probability of supporting the establishment of each proposition during the fusion process, which provides the possibility to change the final conclusion through the orthogonal combination of multiple evidence bodies.

## 5. Application and Analysis of the Evaluation Model of Reformation and Revolution System for Universities Based on Neural Network

### 5.1. Neural Network Input and Output Calculation

It may take issuing open questionnaires, preliminary screening indicators, expert guidance, and generating questionnaires to analyze the reliability and reliability of the questionnaire. The validity adopts rigorous statistical methods and establishes a formal questionnaire evaluation form for the evaluation of the quality evaluation system of revolution and reformation at S University to ensure the smooth development of the next step. One hundred copies of the questionnaire were randomly distributed in the university town where S University is located, and 96 copies were effectively recovered, with an effective recovery rate of 96%.

The valid questionnaire data was input into SPSS, and the reliability and validity of the questionnaire were analyzed. Data normalization is an essential step in the principal component analysis. Since different variable data in the original data are not in the same order of magnitude, if the original data is directly brought into the model without any processing, it will directly affect the model. During neural network training, the selection of initial weights has a great relationship with whether the network learning and training will fall into a local minimum point and whether they will converge. The initial weight is generally a random number, and the value is required to be relatively small. After comparing multiple training processes, the initial weight of the network is selected between [−1/*n*, l/*n*], which can greatly shorten the convergence time.

(14) $$\begin{array}{c} s{\left(i,j\right)}^{t}s\left(i,j\right)=\frac{1-\max s\left(i,j\right)\min s\left(i,j\right)-1}{1+\max s\left(i,j\right)1+\min s\left(i,j\right)}. \end{array}$$

**Figure 5:**
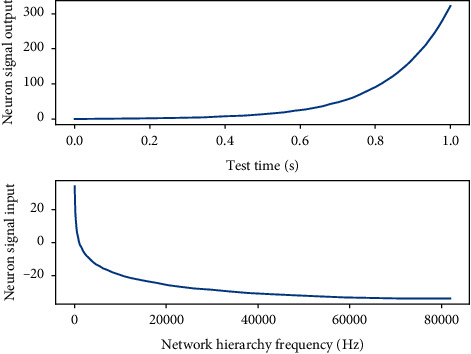
Neuron signal output in the hidden layer of the neural network.

The system consists of two major modules, namely front-end management and back-end management; the functions implemented in B/S mode (front-end management) include user login, password modification, evaluation of colleges and universities, and query evaluation results. User login function: check whether the user name and password entered by the user are correct; if they are correct, the login is successful; otherwise, go to the error prompt page. The indicator system includes 4 first-level indicators and 21 second-level indicators, covering the specific contents of four aspects: student background, student professional ability, student practical ability, and student development ability as shown in Figure 5.

It needs to be normalized before entering the BP network because the values are not the same. The general method of normalization is to set the input as [0, 1]. It can better preserve information to convert the input and output quantities to values in the [0, 1] interval. The input unit acts as the connection window between the network and the outside, and its function is to transmit external data to the inside of the network; although the position of the hidden unit in the entire network structure is not obvious, its function is very important. The internal connection of the network model is its main responsibility; the output unit, as the name suggests, is the output result of the network model, which is the end point of forward propagation and the starting point of reverse transfer. Although weights and thresholds are less discussed, they are also two very important concepts in neural networks.

(15) $$\begin{array}{c} \left[\begin{array}{c} \left(n-1\right)\frac{\partial s\;{\left(i,k\right)}^{t}\partial s\left(i,k\right)}{\partial s\left(i^{\prime },k^{\prime }\right)}o\left(i,k\right)\\ \frac{\partial s\;{\left(i-1,k-1\right)}^{t}\partial s\left(i-1,k-1\right)}{\partial s\left(i-1^{\prime },k-1^{\prime }\right)}o\left(i-1,k-1\right) \end{array}\right]=\left[\begin{array}{c} 1\\ -1 \end{array}\right]. \end{array}$$

In the actual process of modeling time series, we often encounter some special cases between the heteroscedastic functions of the error series of the series, and the common thing is that there is long-term autocorrelation between the function values. The weight is the connection weight between different neurons in each layer, which has the function of storing information, and the size of the threshold is the key factor that determines whether the neurons in each layer can be activated. For such time series, if the ARCH model is still used for fitting, the order of the moving average series will be a large value, which is not conducive to the estimation of the unknown parameters of the model, which will lead to the failure of model fitting. Based on the problems discussed above, the GARCH model takes the *p*-order autocorrelation of the heteroscedastic function into consideration on the basis of the original structure of the ARCH model, thereby weakening the assumptions of the model and strengthening the comprehensive induction ability of the GARCH model, which improves the accuracy of parameter estimation.

### 5.2. Simulation Realization of Reformation and Revolution System in Colleges and Universities

After the above-mentioned neural network training, the weights and thresholds have been determined, and after 16 input values are given, the evaluation result of the neural network can be obtained. The realization of this part is also realized through the design of the evaluation database. Data transformation is performed on the original variables, which reduces the number of variables and eliminates correlations, and at the same time ensures that the transformed new variables can cover principal components, and they are not correlated with each other. The application scenarios of principal component analysis are mostly to remove the multicollinearity between the data before modeling. The analysis result is to obtain a new set of linearly independent comprehensive variables, and the information carried by these variable data is the same as the original variables. The transformation method used between the two sets of variables is the so-called “linear combination” method in mathematics.

(16) $$\begin{array}{c} \left[\begin{array}{cc} \text{at}\left(n-1\right) & w\left(n\right)\\ 1 & -\text{at}\left(n-1\right) \end{array}\right]⟶\left[\begin{array}{cc} \frac{\partial \left(n-1\right)}{\partial n} & \frac{w\left(n\right)}{n}\\ 1 & \frac{-\left(n-1\right)}{n} \end{array}\right],\text{if}\left\{e\left[w\left(n\right)\right]<e\left(0\right)\right\}. \end{array}$$

**Figure 6:**
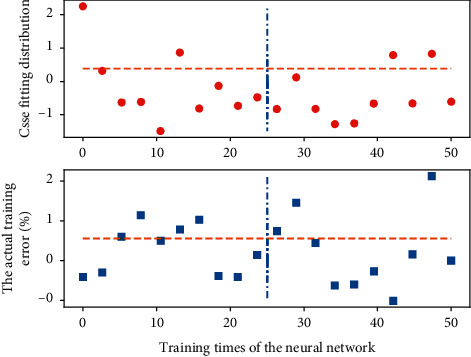
The actual training error fitting distribution of the neural network.

Selecting a representative classroom, formulate a classroom revolution and reformation questionnaire according to the quality evaluation index of revolution and reformation, as the value of the 23 input indicators of the college, so as to eliminate the factors that some students are irresponsible in evaluating colleges and universities. As for the expected output indicators, this paper adopts the evaluation value of the revolution and reformation supervision group in Figure 6. Sample data is a very important link in the training of the neural network, which directly affects the results of network training. Therefore, in the selection of sample data, scientific analysis methods must be adopted to select appropriate samples.

If the learning rate is too small, the convergence speed will be reduced by prolonging the training time; if it is too large, the instability of the system and the interleaving of iterations will appear in the operation process. At the same time, learning rates that perform well at the beginning of training are not suitable for later training. Therefore, the basis for selection can only come from past experience, and this model selects the learning rate range between 0.005 and 0.9. If the number of hidden layers selected is too small, network training cannot be performed, or different samples cannot be identified, and the fault tolerance rate is poor, but if the number of hidden layer units exceeding a certain limit is used, it will prolong the learning time and reduce the neural network convergence speed; the error cannot be minimized. Therefore, there must be an optimal number of hidden layer neurons in a certain BP network.

(17) $$\begin{array}{c} \text{if}\begin{array}{c} e\left[w\left(n\right)\right]>1\\ w\left(n\right)>1 \end{array},\left.\begin{array}{c} \Delta w\left(i,j\right)-\Delta v\left(i,j\right)\\ \Delta w\left(i,j\right)+\Delta v\left(i,j\right) \end{array}\right\}=\int \frac{w\left(i,j\right)}{v\left(i,j\right)}. \end{array}$$

Part of the basic data of this system comes from the educational administration system. Students can evaluate the teaching universities, and experts also need to evaluate the teaching colleges. Therefore, the system must determine who can conduct a certain college based on the course scheduling information and the basic information of the college. For evaluation, the basic data to be used include college information, student information, course information, and class scheduling information. In addition, the system needs to save student evaluation form information and expert evaluation information. This paper does not introduce the data structure from the educational administration system but only introduces the data structure of this system. Through the analysis of the indicators *X4* = 0.84, *X*5 = 0.96, *X*14 = 0.96, *X*34 = 0.92, and *X*36 = 0.90, it can be known that S University has paid attention to encouraging students to study in relevant professional fields while teaching relevant professional courses.

The learning of the neural network is not simply to memorize the learned input but to learn the inherent regularity hidden in the sample through the training sample, so as to give a correct response to the input that does not appear, that is, the generalization of the network. There are two main factors that affect the generalization ability, one is the quality, quantity, and representativeness of the training samples; the other is the neural network itself, including the structure and algorithm of the network. In addition to imparting professional theoretical knowledge, it pays attention to the practice of revolution and reformation and combines the characteristics of relevant professional disciplines, starting from discipline design, internship positions, after-school experiments, and other links for continuous improvement.

### 5.3. Example Application and Analysis

The simulation case analysis in this paper adopts the neural network structure of the input layer, hidden layer, and output layer after training. The number of factors affecting the evaluation result can determine the number. According to the previous indicator system analysis, there are 45 evaluation indicators related to the quality evaluation indicators related to S University, so the input layer node is 45 hidden layer nodes. In order to evaluate students' innovation and entrepreneurship ability, the score corresponding to the student's evaluation index is used as the input vector, the number of neurons in the hidden layer is determined according to the effect of the simulation experiment, and the experimental evaluation result is used as the output vector. The second is to build an evaluation index system for innovation and entrepreneurship education for computer majors.

Fault tolerance and generality will improve test accuracy. The above analysis shows that the number of input layer nodes, hidden layer nodes, and output layer nodes of the structural model, in this case, is 45, 12, 1, and 45 × 12 × 1. The improved neural network model is for S University related majors in reformation and revolution education and revolution. The model was established for reformation quality evaluation. The result of the evaluation is the number of output nodes. In this case, the number of nodes is 1, which is the comprehensive score value of the quality evaluation of reformation and revolution education at S University.

(18) $$\begin{array}{c} \left\{\begin{array}{c} \int p\left(x,y\right)\text{d}x\text{d}y=\sqrt{x-y}-p\left(x\right)p\left(y\right),\\ \int q\left(x,y\right)\text{d}x\text{d}y=\sqrt{x+y}-q\left(x\right)-q\left(y\right). \end{array}\right. \end{array}$$

Colleges and universities can only query the evaluation results of the colleges themselves. Experts can view the evaluation results of all evaluators and print the total evaluation results by the department to each department chair. (1) Colleges and universities can only inquire about their own evaluation results and the evaluation opinions of students, colleges, and experts and have no right to view the evaluation results of others. (2) Colleges and universities can query the scores of individual indicators and the comprehensive scores of the semester for students, colleges, and experts and can also display the comparison between each score and the average score through charts. (3) Experts can inquire about the evaluation results of all evaluators by entering the query conditions such as the academic year, semester, university number, name, department, and so on and display the query results in pages. (4) Print the total assessment status by the department. The background management part and its functional modules include assessor management, data preparation module, neural network training, and result evaluation.

  Step 1: Obtain the financial time series and normalize the input time series data (using the [0, 1] range)
  Step 2: Perform PCA (principal component analysis) dimensionality reduction processing on the normalized data
  Step 3: Select samples and divide them into the training set and the test set, set adjustable constants, error variables, and deviations
  Step 4: Select RBF radial basis kernel function;
  Step 5: After the training set data is dimensionally reduced, the model is to carry out the parameter processing of coefficient group optimization and input the LIBSVM for training to obtain the training function
  Step 6: Input the time series data to be predicted after normalization and principal component dimension reduction into the support vector machine model and perform LIBSVM support vector machine prediction output
  Step 7: Cross-validate and output the results and evaluate the prediction results; if the prediction requirements cannot be met, you need to retrain the model and jump back to Step 3 directly and go to this step again until you are satisfied with the result.

**Figure 7:**
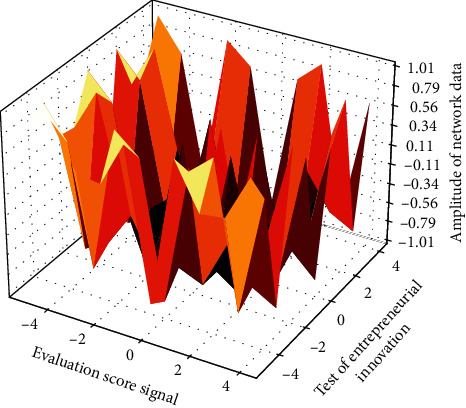
Evaluation distribution of entrepreneurial reformation system under neural network.

MATLAB is also used to normalize the data in [0, 1], and then PCA is used to reduce principal components by 95%. The dimension is reduced from 37 dimensions to 7 dimensions. The PSO coefficient group parameter optimization method is used, and the evolution is set to 200. The number of populations in Figure 7 is 40, and the optimal parameters are obtained. Then the support vector machine selects the RBF kernel function and starts training samples and predicts, and the cross-validation is set to tenfold. The above operations have been repeatedly scrutinized, tried, and verified. The support vector machine model is constructed by the principal component analysis method and coefficient group optimization algorithm, that is, after normalizing the data, the multidimensional data is dimensionally reduced by the principal component analysis method, and then the coefficient group optimization algorithm is applied to it. Increasing the number of hidden layers can reduce the error and improve the accuracy, but it also complicates the network, thereby increasing the training time of the network weights; to improve the accuracy, you can also increase the number of neurons in the hidden layer. In terms of implementation, it is much simpler than adding hidden layers. This conclusion can prove that the principal component analysis method and the GARCH model are more accurate and have good stability. Therefore, it is feasible and effective to apply this model to the evaluation and prediction of the entrepreneurial reformation quality of the entrepreneurial reformation system index.

## 6. Conclusion

This subject takes students of related majors of universities as the research object, combines qualitative analysis with quantitative analysis, and builds a universal related professional reformation and revolution education evaluation system on the basis of the neural network, which reduces the human factors in the evaluation process. The resulting interference ensures the evaluation results. Specifically, this paper has done the following work: (1) a comprehensive introduction is given to the domain knowledge of neural networks, and a systematic study of the construction and training of neural network models is done. (2) The existing problems and improvements of the neural network are introduced, and the improvement method of neural network and simulated annealing is done. On this basis, the network structure, learning parameters, and learning algorithm of the entrepreneurial reformation quality assessment model are determined. (3) The system adopts structure- and object-oriented programming technology to realize the efficient, networked, and intelligent evaluation of entrepreneurial reformation quality. It improves the research and analysis framework in related fields, enriches the research results of reformation and revolution education evaluation in related fields, and has important theoretical value for improving the evaluation model in this field. The evaluation system of teaching quality in colleges and universities is a complex non-linear system, and there are many uncertain factors between input and output. Because of its highly non-linear function mapping function, the artificial neural network model is applied to the teaching quality evaluation system. The above research shows that the system is not only feasible but also the accuracy meets the requirements. The innovative quality assessment model provides a feasible solution.

## Figures and Tables

**Table 1:** Neural network iteration process error.

Prediction node	Iteration 1	Iteration 2	Iteration 3	Iteration 4	Iteration 5	Iteration 6	Iteration 7
1	2.3	2.44	2.58	2.72	2.86	3	3.14
2	1.77	1.87	1.97	2.07	2.17	2.27	2.37
3	1.24	1.3	1.36	1.42	1.48	1.54	1.6
4	0.71	0.73	0.75	0.77	0.79	0.81	0.83
5	0.18	0.16	0.14	0.12	0.1	0.08	0.06

**Table 2:** Evaluation scale of reformation and revolution system.

Evaluation scale index	Fluctuation number	Reformation rate	Residual ratio
ACF^a^	86	60.56	21.22
ACF^b^	39	46.24	21.95
ACF^c^	44	52.65	22.50
GARCH^a^	77	2.26	22.74
GARCH^b^	43	76.90	22.67
GARCH^c^	11	10.94	22.36
GARCH^d^	12	48.93	21.96

“a-d” represent 4 different confidence intervals.

**Table 3:** Recursive description of the neural network training algorithm.

Neural network training code	Recursive description content
Import java.awt.event.actionevent	The selection of *u*+*vt*
String name1 = name.gettext()	*f* ∈ *S* has an important influence
String address1 = address.gettext()	The initial weight *f*(*u*, *t*)
Private jtextfield number = new jtextfield()	SQL Server *z*(*s*, *t* − *s*)
But1.addactionlistener(new actionlistener())	The neural network ∇*h*(*m*, *mt*)
Import java.io.filenotfoundexception	On the training of *g*(*y*, *y*′)
Import java.io.fileoutputstream	For number case between *i*=0,1,2,…, *n*
Hashtable < string, person > has = new < string, person > ()	Developing the (*f*+*j*)(*f* − *j*)
File file = new file(“fiel.txt”)	Quality assessment system *x*(*r*, *t*^*i*^)
Sex.settext(null); date.settext(null)	Entrepreneurial reformation *y* < *y*′

## Data Availability

The data used to support the findings of this study are available from the corresponding author upon request.
